# Clinical and iPSC-based characterization of the HNF1A p.T82M variant

**DOI:** 10.1016/j.gendis.2026.102077

**Published:** 2026-02-13

**Authors:** Ran Liu, Aysha Bakari Murusuri, Siyi Huang, Zhe Dai, Wei Jiang, Jun Tang

**Affiliations:** a Department of Biological Repositories, Frontier Science Center for Immunology and Metabolism, Medical Research Institute, Zhongnan Hospital of Wuhan University, Wuhan University, Wuhan, Hubei 430071, China; b Department of Endocrinology, Zhongnan Hospital of Wuhan University, Wuhan University, Wuhan, Hubei 430071, China; c The Institute of Translational Medicine, The Second Affiliated Hospital, School of Basic Medical Sciences and Institute of Biomedical Innovation, Jiangxi Medical College, Nanchang University, Nanchang, Jiangxi 330031, China

Maturity-onset diabetes of the young (MODY, MIM#606391) is a genetically and clinically heterogeneous group of disorders classified as a form of monogenic diabetes with an autosomal dominant mode of inheritance.^1^ MODY is reported to have a high misdiagnosis rate of approximately 80%, often diagnosed as type 1 or type 2 diabetes mellitus. MODY3 (MIM#600496), also termed HNF1A-MODY, results from heterozygous mutations in the hepatocyte nuclear factor-1-alpha gene (HNF1A) located on chromosome 12q24. The *HNF1A* gene comprises 10 exons encoding a 631-amino acid protein that functions as a crucial transcription factor regulating development and insulin secretion. HNF1A-MODY typically manifests during adolescence or early adulthood with progressive insulin secretory dysfunction and exhibits marked sensitivity to sulfonylureas,^1^ therefore, early diagnosis is critical.

Here, we identified a heterozygous missense variant (c.245C>T, p.Thr82Met [p.T82M]) in exon 1 of the *HNF1A* gene in a MODY-suspected Chinese family. Notably, three of these individuals had been previously misdiagnosed with type 2 diabetes. The clinical manifestations and therapeutic responses exhibited significant heterogeneity among the affected family members. Furthermore, we developed an induced pluripotent stem cell (iPSC) model harboring this specific mutation to investigate its impact on β-cell differentiation.

The proband (II-1; Fig. 1A), diagnosed with type 2 diabetes at age 50, had a family history of diabetes in her son, sister, and brother (Fig. 1A). Clinical and laboratory data are shown in Table S1, including the negative islet autoantibodies. Oral glucose tolerance test (OGTT) of the proband showed the fasting glucose of 7.13 mmol/L and 2-h postprandial glucose of 21.27 mmol/L, while fasting C-peptide of 1.62 ng/mL and 2-h postprandial of 6.12 ng/mL (Table S2). The proband's sister (II-6; Fig. 1A) was diagnosed with type 2 diabetes at age 26, and the OGTT results showed fasting glucose of 8.68 mmol/L and 2-h postprandial glucose of 23.81 mmol/L, with corresponding C-peptide levels of 1.60 ng/mL and 5.84 ng/mL, respectively (Table S1, S2). The proband's son (III-2; Fig. 1A) was diagnosed with type 2 diabetes at age 10, and the OGTT showed diabetic levels of blood glucose with fasting 14.65 mmol/L and 2-h-postprandial 20.7 mmol/L. The fasting insulin was 44.6 μIU/mL, and the 2-h-postprandial insulin was 56.8 μIU/mL (Table S2). The presence of diabetes across two generations, early-onset cases, and negative autoantibodies was suggestive of a MODY phenotype and prompted further genetic evaluation.

**Figure 1:**
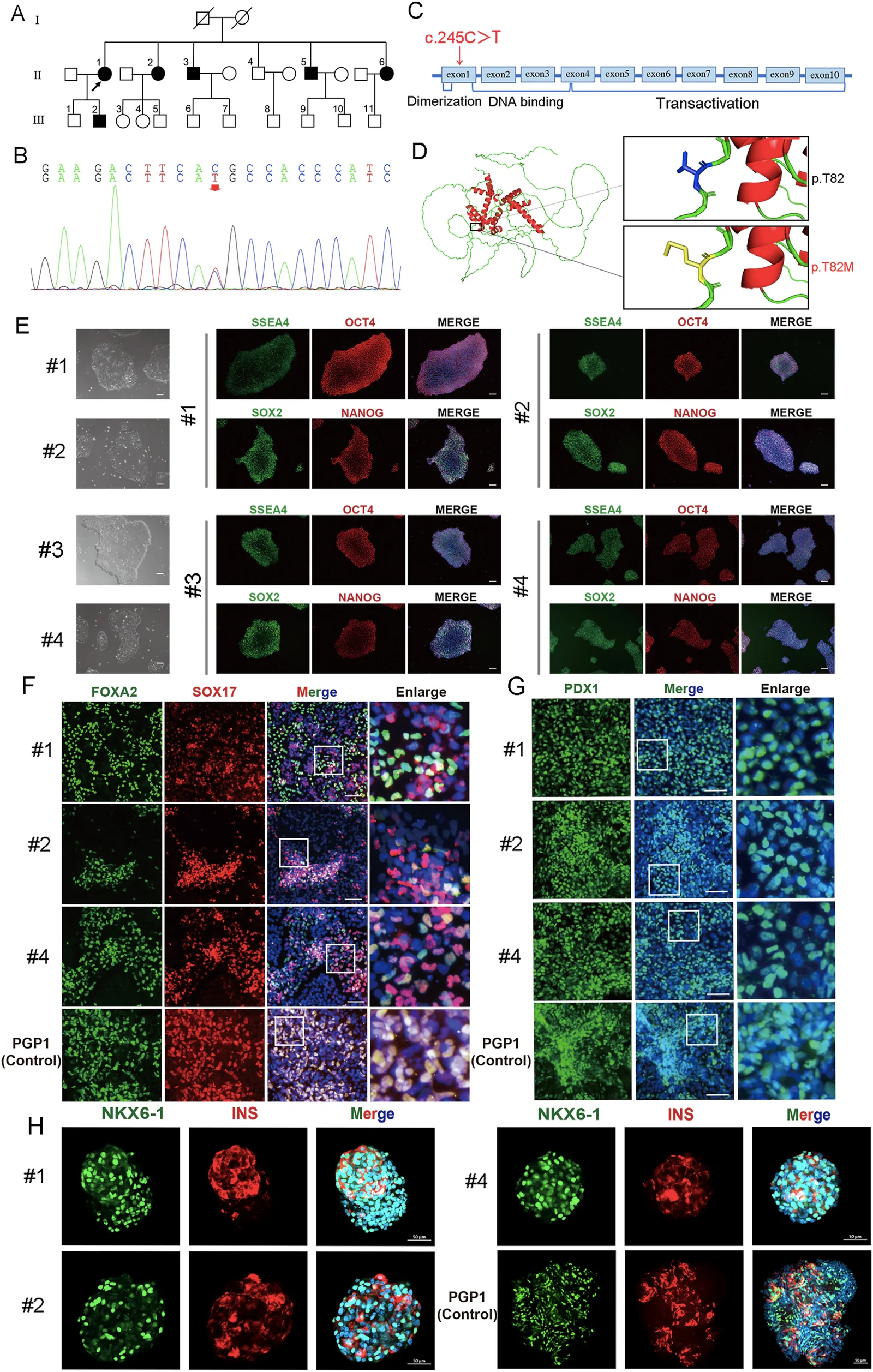
Clinical, genetic, and induced pluripotent stem cell (iPSC)-based characterization of the HNF1A p.T82M variant. **(A)** Family pedigree. The arrow indicates the proband. Deceased family members are marked with a strike-through symbol. The black symbols represent individuals with diabetes, while the blank symbols represent individuals without diabetes. **(B)** Sanger sequencing showing the heterozygous mutation (c.245C>T) in the proband. **(C)** Schematic representation of the HNF1A protein domains. The position of the p.T82M mutation is highlighted. **(D)** PyMOL-generated cartoon model of the HNF1A protein structure based on the SWISS-MODEL. The threonine (T) at position 82 and the mutated methionine (M) are shown as stick models. **(E)** Pluripotent markers of four iPSC lines. Immunofluorescence staining confirmed the expression of pluripotency markers SOX2, NANOG, OCT4, and SSEA4. Scale bar, 100 μm. **(F)** Immunofluorescence staining of definitive endoderm (DE) markers SOX17 and FOXA2. Scale bar, 100 μm. **(G)** Immunofluorescence staining of pancreatic progenitor (PP) marker PDX1. Scale bar, 100 μm. **(H)** Immunofluorescence staining of β-like cells with markers NKX6-1 (green) and Insulin (INS, red). Nuclei were counterstained with DAPI (blue). Scale bar, 50 μm.

Whole-exome sequencing followed by Sanger confirmation identified a heterozygous missense mutation c.245C>T (p.T82M) in exon 1 of the *HNF1A* gene in the proband (Fig. 1B, C). The c.245C>T mutation was detected in II-6 and III-2 as well. This clinical variability is consistent with the known phenotypic spectrum of monogenic diabetes disorders. For the family, the variant was absent in individuals III-4 and III-9, but present in III-11, a 22-year-old man who is currently non-diabetic. This could reflect age-dependent penetrance, which has been reported in HNF1A-MODY, where cumulative diabetes risk increases with age. Other family members declined genetic testing (Fig. S1A). According to the specifications released by the ClinGen Monogenic Diabetes Expert Panel for adapting the ACMG/AMP variant interpretation guidelines to *HNF1A*, the HNF1A variant c.245C>T (p.T82M) could be classified as a variant of uncertain significance based on combined evidence from segregation analysis, computational predictions, and clinical findings (PP1+PP3+PP4). This classification is supported by: i) PP1: co-segregation with diabetes in multiple affected family members; ii) PP3: concordant *in silico* predictions of pathogenicity (REVEL score 0.748); and iii) PP4: clinical presentation consistent with MODY (MODY probability calculator score ≥ 50%, negative pancreatic autoantibodies, and exclusion of *HNF4A* mutations). Post-diagnosis, sulfonylureas were added to II-6's regimen, halving her insulin dose while maintaining glycemic control. III-1 continued SGLT2 inhibitors, and the proband retained her current therapy: acarbose, premeal rapid-acting insulin, and basal insulin.

Using PSIPRED software, we analyzed the secondary structure of the T82M-mutated HNF1A protein (Fig. S1B). Structural analysis revealed significant alterations due to T82M mutation: the native α-helix (residues 86–90) within the POU domain's helix-turn-helix DNA-binding motif was replaced by a β-strand structure (residues 86–88), and a novel helix turn emerged in the previously disordered region (residues 36–37). In addition, the T82M mutation was in an intrinsically disordered region (residues 32–82) and resulted in the addition of a helix turn (residues 36–37) that was previously not present. Disordered regions usually increase protein flexibility in structure and functional capacity, and may indirectly influence binding to or interaction with DNA and other proteins.^2^ Using PyMOL software, we generated a cartoon representation of the HNF1A protein structure based on SWISS-MODEL predictions (Fig. 1D). The model highlights the structural context of residue 82, with both native threonine and mutated methionine side chains displayed as stick models, illustrating the molecular environment of this critical position within the protein's three-dimensional architecture. A previous study assessed the p.T82M variant using luciferase reporter assays in HeLa (human cervical carcinoma) and INS-1 (rat insulinoma) cell lines.^3^ However, the sensitivity of the luciferase reporter is highly dependent on promoter/sequence and the cellular context. Gene expression is precisely but differentially regulated in different cell types, and HeLa and INS-1 cells may not represent the human developmental and physiological conditions. In contrast, patient-derived iPSCs can differentiate into functional, glucose-responsive β-like cells, recapitulating human pancreatic development and β-cell function, thereby providing a more physiologically relevant platform to investigate *HNF1A* variants and diabetes pathogenesis.

Therefore, we collected the peripheral blood mononuclear cells (PBMCs) from II-6 to make patient-specific iPSCs to evaluate whether the HNF1A (c.245C>T, p.T82M) mutation affects β-cell differentiation. After the electroporation with an episomal vector carrying Yamanaka factors^4^, pluripotent-like colonies were isolated at day 21, yielding four iPSC lines (Fig. 1E). These lines expressed typical pluripotent markers including SOX2, NANOG, OCT4, and SSEA4 (Fig. 1E). Sanger sequencing confirmed the heterozygous c.245C>T mutation in all lines (Fig. S2A), while genomic PCR verified the clearance of exogenous episomal plasmid (Fig. S2B), together with the endogenous expression of *NANOG* and *SOX2* (Fig. S2C). Teratoma assays confirmed tri-lineage differentiation potential (Fig. S2D), and G-band analysis revealed normal karyotypes (Fig. S2E). All iPSC lines were confirmed to be free of mycoplasma contamination by commercially available kits. These data together demonstrate that the patient-specific iPSC lines carrying the HNF1A (c.245C>T, p.T82M) mutation are established.

To investigate the potential impact of HNF1A (c.245C>T) on pancreatic development, we employed a directed differentiation protocol of pancreatic β-like cells from human pluripotent stem cells^5^. Differentiation efficiency was assessed using stage-specific markers: FOXA2/SOX17/CXCR4 for definitive endoderm (DE), PDX1 for pancreatic progenitors (PP), and NKX6-1/INS for β-like cells, respectively. Interestingly, all three lines tested carrying this mutation exhibited regular DE formation, as evidenced by comparable FOXA2/SOX17 expression (Fig. 1F; Fig. S2H) and CXCR4-positive cell populations (Fig. S2F). RNA analysis also showed comparable expression of DE markers, including *FOXA2*, *SOX17*, and *GATA6*. In addition, similar PDX1 levels by immunofluorescence and flow cytometric analysis, as well as quantitative reverse transcription PCR, were observed at the PP differentiation stage (Fig. 1G; Fig. S2G, S2I). Similarly, β-like cell differentiation showed comparable NKX6-1/INS expression (Fig. 1H), and quantitative analysis of RNA expression also revealed no statistically significant differences observed for β-cell maturity genes, including *MAFA*, *IAPP*, *PCSK1*, and *PCSK2* (Fig. S2J). These findings indicate that the *HNF1A* (c. 245C>T) variant does not obviously block pancreatic lineage specification or β-cell differentiation. Of note, the potential impact of p.T82M mutation on β-cell function, such as glucose-stimulated insulin secretion, needs to be further evaluated in the future.

In summary, we identified the HNF1A c.245C>T (p.T82M) variant in a Chinese family with a HNF1A-MODY-like phenotype. Based on structural predictions, the variant may potentially impact DNA-binding or transcriptional regulation, though further experimental validation is needed to confirm its functional effects. We further generated the patient-specific iPSC lines, providing a valuable resource for studying molecular mechanisms and developing targeted therapies.

## CRediT authorship contribution statement

**Ran Liu:** Writing – original draft, Visualization, Methodology, Investigation, Formal analysis, Conceptualization. **Aysha Bakari Murusuri:** Writing – original draft, Software, Methodology, Investigation, Formal analysis, Data curation, Conceptualization. **Siyi Huang:** Writing – original draft, Visualization, Resources, Methodology, Formal analysis, Data curation. **Zhe Dai:** Writing – review & editing, Resources, Investigation, Formal analysis. **Wei Jiang:** Writing – review & editing, Validation, Supervision, Resources, Project administration, Methodology, Investigation, Formal analysis, Data curation, Conceptualization. **Jun Tang:** Writing – review & editing, Validation, Supervision, Software, Resources, Project administration, Methodology, Investigation, Funding acquisition, Formal analysis, Data curation, Conceptualization.

## Ethics declaration

This study was approved by the Medical Ethics Committee of Zhongnan Hospital of Wuhan University (No. 2023029). Informed consent was obtained from all parties involved.

## Funding

This study was supported by the Discipline Cultivation Funding, Zhongnan Hospital of Wuhan University (China) (No. ZNXKPY2022031) and Translational Medicine and Interdisciplinary Research Joint Fund of Zhongnan Hospital of Wuhan University (China) (No. ZNJC202513).

## Conflict of interests

The authors declared no conflict of interests.
