# Supplementary material for: Clinical and iPSC-based characterization of the HNF1A p.T82M variant
Source: Genes Dis. 2026 Feb 13;14(1):102077. doi: 10.1016/j.gendis.2026.102077 (PMC13571237; doi:10.1016/j.gendis.2026.102077)
Supplement: Supplementary file 1 [file mmc1.docx]

**Supplementary Materials**

**Figure S1. Sanger sequencing of the HNF1A p.T82M mutation and its structural implications.**

(A) Sanger sequencing of the HNF1A p.T82M mutation. (B) Analysis of HNF1A and variants in secondary structure.

**Figure S2. Genetic validation, pluripotency, and differentiation analysis of patient-specific iPSCs.**

(A) Sanger sequencing confirming the presence of the heterozygous c.245C>T mutation (p.T82M) in the HNF1A locus across the four iPSC lines. (B) Genomic PCR analysis demonstrating the absence of episomal plasmid integration in the iPSCs. (C) RT-PCR analysis showing the endogenous expression of NANOG and SOX2 in the iPSCs. (D) Representative tissues of all three germ layers from the teratoma assay of iPSCs. Scale bar, 100 µm.

(E) G-band karyotype analysis, confirming normal karyotype and genomic stability. (F) Flow cytometry analysis and quantitative analysis of CXCR4 expression at DE stage (n=3). (G) Flow cytometry analysis and quantitative analysis of PDX1 expression at PP stage (n=3). (H-J) Quantitative analysis of key marker gene RNA expression during the DE phase, PP phase, and β-like cell phase (n=3).

**Table S1: Clinical Characteristics and Laboratory Findings of the patients**

|  | II-1 | II-6 | III-2 |
| --- | --- | --- | --- |
| Born | 1961 | 1979 | 1993 |
| Diabetes onset | 2011 | 2005 | 2003 |
| Age of diabetes onset (years) | 50 | 26 | 10 |
| Height | 160 | 164 | 180 |
| Weight | 59 | 68 | 90 |
| BMI (kg/m^2^) | 23.05 | 25.28 | 27.77 |
| HbA1C (%) |  | 8.2 | 9.2 |
| Anti-insulin Ab | Negative | Negative | Negative |
| GAD-Ab | Negative | Negative | Negative |
| Total cholesterol (mmol/L) | 4.58 | 6.32 | 5.8 |
| Triglycerides (mmol/L) | 1.09 | 2.37 | 6.47 |
| HDL-C (mmol/L) | 1.37 | 1.19 | 0.94 |
| LDL-C (mmol/L) | 2.79 | 3.76 | 2.76 |
| Creatinine (µmol/L) | 69.4 | 59.5 | 66.8 |
| Uric acid (µmol/L) | 302.2 | 424.4 | 596.2 |
| Hypertension | Yes | No | Yes |
| Coronary heart disease | No | Yes | No |
| Diabetic chronic complications | DR, DPN | DPN | DPN |

BMI: body mass index; Ab: antibody; GAD: glutamic acid decarboxylase

HDL-C: high density lipoprotein cholesterol; LDL-C: low density lipoprotein cholesterol

DR: diabetic retinopathy; DPN: diabetic peripheral neuropathy

**Table S2: OGTT, C-peptide, and insulin levels of the patients**

|  | **II-2** | **II-6** | **III-2** |
| --- | --- | --- | --- |
| Blood glucose (mmol/l) | |  |  |
| 0 min | 7.13 | 8.68 | 14.65 |
| 30 min | 14.74 | 16.54 | 19.09 |
| 60 min | 19.24 | 19.61 | 19.88 |
| 120 min | 21.27 | 23.81 | 20.7 |
| 180 min | 17.58 | 18.73 | 17.94 |
| C-peptide (ng/ml) |  |  |  |
| 0 min | 1.623 | 1.599 | - |
| 30 min | 2.454 | 2.837 | - |
| 60 min | 4.5 | 3.93 | - |
| 120 min | 6.117 | 5.841 | - |
| 180 min | 4.965 | 6.212 | - |
| Insulin (mIU/mL) |  |  |  |
| 0 min | 9.302 | 12.52 | 44.6 |
| 30 min | 18.81 | 27.2 | 59.6 |
| 60 min | 27.28 | 32.22 | 57.6 |
| 120 min | 41.05 | 38.13 | 56.8 |
| 180 min | 35.79 | 39.02 | 55 |
