# Supplementary material for: Clinical and iPSC-based characterization of the HNF1A p.T82M variant
Source: Genes Dis. 2026 Feb 13;14(1):102077. doi: 10.1016/j.gendis.2026.102077 (PMC13571237; doi:10.1016/j.gendis.2026.102077)
Supplement: Supplementary file 2 [file mmc2.docx]

**Materials & methods**

**Clinical studies**

This study was approved by the Medical Ethics Committee of Zhongnan Hospital of Wuhan University (No: 2023029). Informed consent was obtained from all parties involved. Clinical data were collected, including current medical history, family history, laboratory test results, and other relevant medical information.

**Genetic testing**

Peripheral blood samples (2 mL) were collected from the study subjects and sent to Kangxu Medical Laboratory Co., Ltd. in Beijing for whole-exome sequencing (WES). Genomic DNA was extracted from the blood samples using the Qiagen FlexiGene DNA Kit (Catalog No. 51206, Qiagen, Germany). The DNA samples were fragmented using a sonicator to obtain 150-300 bp DNA fragments. Adapters were added to both ends of the fragmented DNA, and sticky ends were blunted, followed by library amplification and purification through PCR. The DNA from the amplified library was hybridized and captured using probes from the Agilent SureSelect Enrichment System, and then purified and quantified. The enriched DNA was subjected to paired-end sequencing on the Illumina NextSeq500 platform to generate FASTQ format data. Align analysis, SNP analysis, and DIP analysis were performed on the FASTQ data to obtain information on mutations in the targeted regions. The gene sequences of these mutation sites were retrieved from the GenBank human genome database. After PCR amplification of the mutation sites, Sanger sequencing was performed, and the obtained sequences were compared with the previous sequences to eliminate false positives from the next-generation sequencing.

**Bioinformatic analysis**

The variants sites, types, frequencies, and other information were obtained through databases and transcript annotation databases, such as 1000 Genome, Human Gene Mutation Database (HGMD) and the Exome Aggregation Consortium (ExAC). The second structure of protein was predicted by PSIPRED (<http://bioinf.cs.ucl.ac.uk/psipred>). The structure of protein was predicted by SWISS-MODEL (<https://swissmodel.expasy.org/interactive>). PyMOL-generated cartoon model of the HNF1A protein structure based on the SWISS-MODEL.

**Reprogramming of PBMCs**

5 ml blood from the patient were donated with informed consent performed at Zhongnan Hospital of Wuhan University. The genotype with the mutation in HNF1A (c.245C＞T, also p.T82M) was identified by previous next-generation sequencing and further confirmed by PCR followed by Sanger sequencing. The patient-derived PBMCs were isolated by Lymphoprep (STEMCELL Technologies, Cat# 07801), and cultured in StemSpan™ SFEM II medium (STEMCELL Technologies, Cat# 09605) supplemented with CD34+ Expansion supplement (STEMCELL Technologies, Cat# 02691) to expand for 5 days. Subsequently, the cells were electroporated (Amaxa Nucleofector 2B) with four episomal plasmids (addgene # 27076, # 27078, # 27080 and #37624) and then cultured in a 12-well plate coated with Matrigel (Corning, Cat# 354277) in StemSpan™ SFEM II medium. On day 6, medium was changed to reproTeSR (STEMCELL Technologies, Cat# 05920). After another 5 days, medium changed to mTeSR1 (STEMCELL Technologies, Cat# 85850). At about day 21, iPSC clones with typical colony morphology were isolated.

**Cell culture conditions**

IPSCs were maintained in 6-well plates coated with Matrigel in mTeSR1 in a 37 ◦C incubator with a humidified atmosphere of 5% CO_2_. We used Versene (Thermo Fisher Scientific, Cat# 15040066) to split the clones as the ratio of 1:5–1:8. The medium was changed every day.

**Karyotype analysis and short tandem repeat (STR) analysis**

The karyotype analysis of both iPSC lines at about passage 15 were assessed by the Nuwacell Biotechnologies Co., Ltd. About 20 cells metaphase for each line were counted and analyzed. The STR analyses of the iPSC lines were performed by the Nuwacell Biotechnologies Co., Ltd as well.

**Immunofluorescence staining**

Cells were washed with PBS and fixed in 4% paraformaldehyde for 20 minutes at room temperature and then were blocked and permeabilized by block buffer (PBS contain 10% donkey serum and 0.3% TritonX-100) for 2 hours at room temperature after PBS washing. Primary antibodies and secondary fluorescent antibodies were diluted in block buffer at proper concentration. Cells were incubated with diluted primary antibody overnight at 4 °C. After washing with PBS three times, and cells were incubated with diluted corresponding secondary fluorescent antibodies and incubated at room temperature in the dark for 2 hours. The nuclei were counterstained with 2‰ 4,6-diamidino-2-phenylindole (DAPI, Sigma, Cat#10236276001). The results of immunofluorescence were visualized and imaged under an Olympus IX53 microscope. The antibodies for immunofluorescence assay used in present work include: anti-OCT4 (1:200, Cell Signaling Technology, Cat# 2750S), anti-SSEA4 (1:200, Millipore, Cat# MAB4304), anti-NANOG (1:200, Cell Signaling Technology, Cat# 4903S), anti- SOX2 (1:50, BD Biosciences, Cat# 561469), anti-SOX17 (1:200, R&D, Cat# AF1924), anti-FOXA2 (1:200, R&D, Cat# AF2400). anti-PDX1 (1:200, R&D, Cat# AF2419), anti-NKX6-1 (1:500, Cell Signaling Technology, Cat# 54551S) and anti-INS(1:500, DSHB, Cat# GN－ID4).

**Teratoma assay**

2 million dispersed iPSCs were collected and resuspended with 100 μL Matrigel, and then injected into the inguinal groin of BALB/C-nude mice. Teratomas were formed at about 8 weeks, and collected for paraffin section and hematoxylin and eosin staining.

**DNA and RNA analysis**

Genomic DNA of the iPSCs were isolated with HiPure Tissue DNA mini Kit (Magen, Cat# D3121-02). Total RNA from cells was extracted and purified using HiPure Total RNA Mini Kit (Magen, Cat# R4111-03). 1ug of extracted RNA was reverse-transcribed into cDNA using AB Script II RT master Mix (Abclonal, Cat# RK20402).

**Cell culture and differentiation**

For pancreatic β cell differentiation, a published differentiation protocol and our previous work^1;2^ were used here with certain modifications. Briefly, human PSCs were isolated into single cells using Accutase (STEMCELL Technologies, Cat# 07922) and seeded at growth-factor-reduced Matrigel-coated plates with 100,000 to 1250,000 cells/cm^2^ density. For induction of definitive endoderm, plated cells were cultured in MCDB131 (Gibco, Cat# 10372019) supplemented with 1xB27 (Yuanpei, Cat# S441J7), 1 × GlutaMAX(Gibco, Cat# 35050061), 1% PS,0.25 mM ascorbic acid (Gibco, Cat# A5960), 4.5 mM glucose (Sigma, Cat# G7528), 100 ng/mL Activin A (PeproTech, Cat# 120-14 P) ,50 nM PI-103(Selleck, Cat# S1038),10 μM Y27632 2HCL (Selleck, Cat# S1049) and 6 μM CHIR99021 (Selleck, Cat# S2924) for 1 day. The CHIR99021, PI-103 and Y27632 2HCL were removed from the medium for the following 3 days. For induction of Primitive Gut Tube (PGT), obtained DE cells were cultured in MCDB131 (Sigma, Cat# 10372019) supplemented with 1xB27, 1 × GlutaMAX, 1% PS,0.25 mM ascorbic acid (Gibco, Cat# A5960), 4.5 mM glucose (Sigma, Cat# G7528), 50 ng/mL KGF (PeproTech, Cat# 100-19-500), 5 μM SB431542 (Selleck, Cat# S1067) and 100 nM WNT-C59 (Selleck, Cat# S7037). For induction of PDX1+ pancreatic progenitors (PP1), obtained PGT cells were cultured in DMEM (Gibco, Cat# 11965092) supplemented with 1xB27,1% PS, 0.25 μM SANT1 (Selleck, Cat# S7092), 100 nM WNT-C59, 2 μM RA (Sigma, Cat# R2625) and 100 nM LDN193189 (Selleck, Cat# S7507). For induction of PDX1+/NKX6-1+ pancreatic progenitors (PP2), obtained PP1 cells were cultured in DMEM supplemented with 1xB27,1 × GlutaMAX,1% PS, 0.25 mM ascorbic acid,100 ng/mL EGF (PeproTech, Cat# AF-100-15), 0.25 μM SANT1,200 nM PDBU (Sigma, Cat# P1269) and 10 mM Nicotinamide (Sigma, Cat# N0636-500G). For induction of pancreatic endocrine progenitors (PEP), PP2 cells were cultured in MCDB131 supplemented with 1xB27,1xGlutaMAX,1% PS, 0.25 mM ascorbic acid, 10 μM Repsox (Selleck, Cat# S7223), 10 μg/mL heparin (Selleck, Cat# S1346), 10 μM Y27632 2HCL, 100 nM WNT-C59, 300 nM LDN193189, 1 μM T3 (Sigma, Cat# 64245) and 100 nM YO-01027 (Selleck, Cat# S2711). For induction of mature β cells, obtained PEP cells were cultured in MCDB131 with1xB27,1% PS, 0.25 mM ascorbic acid. In addition, 1 μM T3, 10 μM Repsox, 10 μg/mL heparin, 0.5 μM R428 (APExBIO, Cat# A8329), 10 mM N-cys (Sigma, Cat# A9165) and 10 μM zinc sulfate were added for 2 days.

**Flow cytometry**

Cells were digested into single cells by Accutase (STEMCELL Technologies, Cat# 07922) and suspended in PBS containing 2% FBS. Cells were collected by centrifugation at 300 g for 3 minutes at 4 °C. For the detection of the surface marker CXCR4, cells were incubated with CD184-APC (1:200, BD Pharmingen, Cat# 555976) in the dark for 30 minutes at 4 ℃. The corresponding isotype control antibody was used as a negative control. CXCR4-positive cells were analyzed with a flow cytometer (CYTOFLEX). For intracellular antibody staining, cells were re-suspended and fixed according to the manufacturer’s instructions of Transcription Factor Buffer Set (BD, Cat#562574). Fixed cells were washed and then incubated with diluted primary antibodies (anti-PDX1,1:500, R&D, Cat# AF2419) overnight at 4 °C. The cells were then washed and incubated with diluted secondary fluorescent antibodies in the dark for 2 hours at room temperature. Both primary and secondary fluorescent antibodies were diluted in 1x Perm/Wash solution. After washing, cells were resuspended in PBS and analyzed with a flow cytometer (CYTOFLEX).

**Reference**

1.	Tan M, Jiang L, Li Y, Jiang W. Dual Inhibition of BMP and WNT Signals Promotes Pancreatic Differentiation from Human Pluripotent Stem Cells. *Stem Cells Int.* 2019;2019:5026793.

2.	Li Y, Zheng R, Jiang L, et al. A noncoding variant confers pancreatic differentiation defect and contributes to diabetes susceptibility by recruiting RXRA. *Nat Commun.* 2024;15(1):9771.
